# Emergency departments in The Netherlands: is there a difference in emergency departments with and without emergency physicians? a cross-sectional web-based survey

**DOI:** 10.1186/1865-1380-6-11

**Published:** 2013-04-15

**Authors:** Wendy AMH Thijssen, Jan Koetsenruijter, Paul Giesen, Michel Wensing

**Affiliations:** 1 Catharina Hospital, Eindhoven, Michelangelolaan 2, PO box 1350, 5602ZA Eindhoven, the Netherlands; 2 IQ Scientific Institute for Quality of Healthcare (IQ healthcare), Radboud University Nijmegen Medical Centre, PO Box 9101, IQ healthcare 114, 6500 HB Nijmegen, the Netherlands

## Abstract

**Background:**

There is a growing interest in emergency departments (EDs) and the development of emergency medicine in The Netherlands. In the last decade several policy reports have stated that the quality of emergency care should be improved and that emergency physicians (EPs) play a large role in the quality improvement. The Netherlands Society of Emergency Physicians (NVSHA) has developed an emergency medicine training program, which has been nationally recognized since 2009. Nevertheless, not all EDs are staffed with EPs yet. This study aimed to explore differences between Dutch EDs with EPs and those without EPs.

**Methods:**

A cross-sectional web-based survey was performed on data over the year 2008 or 2009 in all 105 Dutch hospitals with an emergency department. We documented which ED-specific courses were attended by physicians working in the ED (list of 3 courses) and which clinical audit activities were implemented (list of 6 activities). The choice of courses and clinical audits was based on those mentioned in published quality reports and in national debates on emergency care. We compared EDs with and without EPs. The final analysis was based on a linear regression analysis, controlling for ED size and having an EP training program. We considered *P* < 0.05 significant.

**Results:**

Our survey’s response rate was 67%. EPs worked significantly more often in larger EDs. The linear regression analysis shows that the total number of courses attended by physicians was on average 0.51 higher (*P* = 0.000) in EDs with EPs than in EDs without EPs, and the total number of implemented clinical audits was on average 0.49 higher (*P* = 0.008). After controlling for potential confounders, the effect of both the composite number of courses attended (*P* = 0.001) and the composite number of implemented clinical activities (*P* = 0.032) remained significant.

**Conclusion:**

This study shows that EPs are significantly more present in larger EDs and in EDs where there is more continuing professional education and where there are more clinical audit activities. Our findings suggest that the presence of emergency physicians is positively associated with the quality of emergency care, but prospective research is required to examine causality.

## Background

Organizing emergency care is an important topic in many countries. Due to rising healthcare costs and the need to improve the quality of emergency care in The Netherlands, there is a nationwide special interest in Dutch emergency departments (EDs) and the ongoing development of emergency medicine. The Inspectorate of Healthcare published several reports stating that the quality of emergency care should be improved and that EDs could be divided into three different categories, ranging from basic EM care in smaller hospitals, to more specialized care in larger teaching hospitals, to full EM care in university medical centers and trauma centers. This has not been implemented during the timeframe of this study. This also is the case for the national Inspectorate of Healthcare’s preference of having an emergency physician (EP) working in every ED [1-3]. In response to these reports, expert consensus panelists developed a national quality requirement framework (QFR) intended to improve EM care in Dutch EDs [4]. A small sample study showed that none of the Dutch EDs complied with these developed indicators. In the first years of the millennium, five Dutch hospitals started an emergency medicine (EM) training program, which has been officially recognized as a candidate specialty, as of January 2009 [5,6]. The main reason for this development was that up until then Dutch emergency departments (EDs) were mostly staffed with young inexperienced physicians having just graduated as doctors [7]. Specialists who were not physically present in the ED supervised these physicians. The Dutch emergency medicine training program is 3 years long and consists of multiple rotations, with intensive care medicine, anesthesiology, cardiology, pediatric medicine, family medicine and ambulance medical services as compulsory. More than 50% percent of the training program takes place in the ED, and there are three compulsory courses: Advanced Trauma Life Support (ATLS), Advanced Life Support (ALS) and Advanced Pediatric Life Support (APLS). Although the literature states otherwise, some medical specialties in The Netherlands still question the need for an Emergency Medicine specialty [4,8-17]. Despite the rapidly growing number of EPs in the Dutch EDs, there are still EDs that do not have EPs. This makes the Dutch setting an ideal place for research. We hypothesized that having an EP in the ED will lead to an improvement in the quality of emergency care. The EP is continuously working in the ED, having EM as his domain, and is therefore more committed to implementing quality improvement measures in the ED than physicians who occasionally visit the ED for patient care [17]. The aim of this study was to look at features of all Dutch hospitals and their emergency departments in general and compare EDs with and without EPs. We also documented the continuing professional education of clinical ED staff through attended courses as well as clinical audits through ED registrations and meetings, which we considered proxy measures of the quality of emergency care.

## Methods

### Design

We performed a cross-sectional web-based survey using Limesurvey (http://www.limesurvey.org). Data of the survey were coded and analyzed in SPSS 18. The primary outcomes of the study were continuing professional education and clinical audit activities in emergency departments. The medical ethics committee of Arnhem-Nijmegen granted institutional review board exemption.

### Population

We obtained an updated list of all hospitals in The Netherlands from the national website http://www.ziekenhuis.nl in December 2009. Of the 170 listed locations, 105 hospitals met the inclusion criteria of having an operating emergency department in the years 2008 and 2009. Between January and March 2010, these 105 emergency departments were contacted by telephone. The purpose was to identify a contact person in each ED, typically the head of staff and preferably the consultant, or the head manager of the ED, to explain the goal of this study. Of the 105 hospitals with an operating ED, 101 agreed to participate. Four EDs refused because the data requested was not available or because of lack of time or interest. A few hospitals had more then one ED location. Where hospitals had an operating ED in more than one location, only one questionnaire was filled out. This was because policies on courses and registrations were the same in multiple ED locations of the same hospital. However, differences between these EDs in general features and registrations that could not be filled out in one questionnaire were separately documented by personal telephone interviews and put in the SPSS database manually. This was also the case if, in the timeframe of the data collection, there had been a change in course policy or registration. A total of 97 questionnaires were eventually sent, representing all of the 101 participating EDs.

### Measures

First, we measured general characteristics of both the hospital and the ED, including the number of hospital beds, acute care facilities, general facilities, number of ED visits per year, percentage of self-referrals and whether EDs used a triage system. Secondly, we checked if an ED had an EP. Having an EP in the ED was defined as having an ED with at least one EP in the group of EDs with EPs. Since EM is a young specialty in The Netherlands, there are not enough EPs to facilitate 24/7 emergency care in all EDs in The Netherlands. We defined Emergency Physicians as physicians who successfully finished the Dutch EM training program and were registered as EPs in the Dutch Medical Specialist Registration Committee (MSRC) or EPs working in the studied EDs that were registered as EPs in their own country with a recognized EM training program. Thirdly, we documented which ED-specific courses physicians needed to have attended, past or present, when working in their current ED (list of 3 courses) and which clinical audit activities were implemented (list of 6 activities). The choice of courses and clinical audits was based on those mentioned in published Dutch emergency department’s quality reports and in national debates on emergency care. The questionnaire was designed by experienced researchers, authors 3 and 4, after reviewing the literature and a pilot testing by a randomly chosen ED. This ED later also participated in the final survey. Each contact person was asked to answer the questions with the most recent data available, preferably over 2009, but if that was not possible then over 2008. It was suggested that filling out the survey could be a joint effort of medical personnel and management working in the ED. This was to minimize the influence of recall bias and missing data. Over a 3-month period, a maximum of three reminder emails were sent if the survey had not been completed. On follow-up, reasons for not filling out the survey were: the available data was incomplete, there was a lack of time or the head of staff had changed during the timeframe of the survey.

### Analysis

For the comparison of characteristics of EDs with EPs and EDs without EPs, we used either *t*-tests or chi-square tests as appropriate. For exploration of a possible effect of the presence of EPs on continuing professional education and clinical audit activities, we used linear regression analysis. This allowed controlling for potential confounders. Dependent variables in this analysis were the number of emergency care courses physicians attended and the number of clinical audit activities implemented. The independent variable was the presence of an EP. We controlled for the size of the ED and the presence of an EP training program. In model I we checked the effect of the presence of EPs on each of these two outcomes, and in model II we added the two potential confounders. Size of the ED was added to control for possible financial and organizational advantages of larger EDs. The existence of an EP training program was added because two of the courses (ALS and ATLS) documented are compulsory in the EM training program. In all analyses we considered *p* < 0.05 as statistically significant. Standard errors in the regression analysis were adjusted for the finite population correction (69 out of 105 EDs in The Netherlands).

## Results

Out of the 97 questionnaires sent, representing 101 EDs, we received 65 questionnaires, a response rate of 67%. These 65 questionnaires represented 69 EDs (67% of all Dutch EDs) (Table 1). The responding hospitals included academic hospitals, trauma centers, large community hospitals and smaller rural hospitals with a mean of 456 (200–1100) beds for hospitals with EPs and 349 (144–600) beds for hospitals without. Every region in the country was represented. Almost all hospitals (over 95%) had an intensive care, a coronary care and a stroke unit. Half of the responding hospitals, the majority in the EP group, reported to have an emergency cardiac care unit where patients with a cardiac diagnosis, or a high suspicion of one, were seen directly by cardiologists, thereby mostly bypassing the emergency department. Overall, we found no significant differences in general hospital features between EDs with EPs and EDs without EPs.

**Table 1:** General descriptions of hospitals and EDs*

			**EP (*N *= 38)**	**No EP (*N *= 27)**	***P *value**
**Hospital features**	Number of hospital beds#		456 (200–1100)	349 (144–600)	0.059
	Distance to the nearest other hospital (km)#		18.3 (2–40)	22 (1–80)	0.332
	Location§	City	15	10	0.494
		Urban	13	12	
		Rural	10	4	
	Type^§^	Academic ^	5	0	0.124
		Trauma center	1	1	
		General	28	25	
	Facilities^§^	Intensive care	38	25	0.329
		Coronary care	38	24	0.133
		Stroke unit	38	25	0.329
		Emergency cardiac care	20	12	0.690
		Acute admission ward^$^	3	0	0.371
**ED features**	Patients per year#		24.613 (7,818–48,230)	19,408 (8,100–42,000)	0.048*
	ED admissions per year#		6,221 (2,100–10,800)	5,418 (2,000–9,975)	0.287
	Self referrals (%)#		34 (0–80)	29 (5–65)	0.427
	Facilities^§^	Open 24/7	38	25	0.847
		Triage system	34	23	1.000
		Shock room	38	20	0.004*
		Observation unit	8	9	0.410

The table describes 65 questionnaires representing 70 hospitals. ^All academic hospitals are also trauma centers. ^$^Chi-square for independence, #independent samples *t*-test, *statistically significant at *P* < 0.05.

Emergency department features show that EDs with EPs had significantly more patients attending the ED per year, with a mean of 24.613 (7,818–48,230) compared to 19.408 (8.100−42.000, *P* = 0.048). We found a mean self-referral rate of 34% (0%−80%) in EDs with EPs compared to 29% (5%−65%) in EDs without EPs. Apart from one missing variable, all emergency departments except one were open 24 h. There was a significant difference for having a shock room in the ED, namely 38 (100%) in EDs with EPs and 20 (74%) in EDs without EPs (*P* = 0.004).

Table 2 shows that physicians had attended significantly more courses in EDs with EPs. Differences, apart from the obvious EP specialty training (50.0% vs. 0.0%, *P* = 0.000), were found for the ATLS course (97.4% vs. 74.1%, *P* = 0.004) and the ECG course (21.1% vs. 11.1%, *P* = 0.023). Radiology meetings, where diagnostic research is reviewed the following day by a radiologist to reduce the number of missed diagnoses, were significantly more implemented in EDs with EPs (97.4% vs. 77.8%, *P* = 0.012). There were no differences for the other clinical audit activities.

**Table 2:** Training of physicians and quality-improving activities in EDs with EPs compared to EDs without EPs

		**Number of emergency departments**		
		**With EP *N *= 38 (%)**	**Without EP *N *= 27 (%)**	***P *value**
**Continuing professional education for physicians**	EP specialty training**	19 (50.0)	0 (0.0)	0.001*
	Advanced trauma life support	37 (97.4)	20 (74.1)	0.005*
	Advanced cardiac life support	38 (100)	25 (92.6)	0.088
	Electrocardiographic course	19 (50.0)	6 (22.2)	0.023*
**Clinical audit activities**	Complications registration^	11(28.9)	5 (18.5)	0.336
	Complaint registration^^	28 (73.7)	19 (70.4)	0.769
	Adverse event reporting^^^	37 (97.4)	25 (92.6)	0.366
	Electronic patient record	29 (76.3)	15 (55.6)	0.078
	Radiology meeting^$^	37 (97.4)	21 (77.8)	0.012*
	Child abuse meeting	37 (97.4)	25 (92.6)	0.366

*Significant. **Not all EDs where EPs work also have an EP specialty training program; ^complications that happened around patient treatments, registered by medical staff; ^^complaints made by patients to the hospital; ^^^serious complications that have to be reported to the inspectorate of health. ^$^A meeting with the EP and a radiologist looking at all radiology diagnostic tests of patients who visited the ED the day before.

The linear regression analysis is shown in Table 3. Model I shows that the total number of courses attended by physicians was on average 0.51 higher (*P* = 0.000) in EDs with EPs than in EDs without EPs, and the total number of implemented clinical audits was on average 0.49 higher (*P* = 0.008). After controlling for potential confounders (model II), the effect of both the composite number of courses attended (*P* = 0.001) and the composite number of implemented clinical activities (*P* = 0.032) remained significant.

**Table 3:** Results of the regression analysis

		**Model I**			**Model II**		
		**B**	**SE**	**P**	**B**	**SE**	**P**
**Continuing professional education for physicians (Mean number of courses attended by physicians)**	Constant	1.96	0.07		1.75	0.13	
	EP (no EP ref.)	0.51	0.10	0.000*	0.37	0.11	0.001*
	No. attending ED patients (× 10,000)				0.11	0.06	0.057
	EP training program				0.16	0.14	0.268
**Clinical audit activities ** **(Mean number of activities)**	constant	5.12	0.14		4.52	0.24	
	EP (no EP ref.)	0.49	0.18	0.008*	0.46	0.21	0.032*
	No. attending ED patients (× 10,000)				0.31	0.11	0.005
	EP training program				−0.28	0.27	0.304
**N**	64						

## Discussion

Our survey’s response rate was 67%. EPs worked significantly more often in larger EDs. We found that in EDs with EPs, physicians attended significantly more courses and implemented more clinical activities than EDs without EPs. These differences remained significant in the regression analyses for both the number of courses and implemented clinical activities.

The lowest number of patient visits per year in our study, representing 69 EDs, was 7,818. The study of Ikkersheim, conducted in the same period and representing 27 Dutch EDs, found a minimum of 3,466 patient visits per year [4]. This could be due to the different percentage of rural hospitals in the overall study, being 21.5% in our study compared to 63% in Ikkersheim’s. They also found a 100% score in having a triage system compared to our 85% in EDs without EPs (100% in EDs with EPs). This could simply be explained by the overall numbers of EDs studied.

Our data also shows that EPs work in small and large hospitals as well as in academic settings in all regions of The Netherlands. EPs however work significantly more often in EDs with larger patient visits per year. This is probably because EPs initially started working in larger EDs with EM training programs and over time expanded to academic hospitals and rural areas. In emergency departments with EPs, physicians had attended more courses both individually and as composite numbers compared to emergency departments without EPs. After controlling for a training program, the number of courses attended remained significant.

We also found that in emergency departments with EPs, more clinical audit activities were undertaken. This association remained significant after controlling for the size of the department. It is likely that the significant difference in the radiology meeting could be the underlying cause for the significant composite numbers of clinical audits in EDs with EPs. A large liability insurance company for hospitals instigated the radiology meeting together with EPs from The Netherlands Society of Emergency Medicine (NVSHA) [18]. The significant difference in the number of hospitals with EPs that have a radiology meeting, suggests the influence of EPs on implementing clinical audit activities. Although the financial capacity for larger EDs might be influential in attending more courses or implementing clinical audits, our data does not support this hypothesis. Very little has been reported on training courses and quality-improving activities in Dutch emergency departments. Van Geloven found a very low percentage of physicians in the ED having followed courses like ATLS (27%) or ECGs (6%). That study however, was done in 1999 before the introduction of the emergency medicine training program [7]. This could explain the higher numbers we found. Our study suggests that EPs have a positive influence on physicians attending ABCDE courses and in implementing clinical audit activities, but it is likely that ED leadership or hospital management is influential as well. Although this was one of the first nationwide inventories of its kind in The Netherlands, and other factors were mentioned as well, the positive influence of EPs suggests that their presence could improve the quality of care and therefore patient safety.

At the moment there are not enough EPs to staff all the EDs in The Netherlands and the length of their training program, 3 years, is not meeting the criteria of the ‘Doctors’ Directive of the European Union, which states it should be 5 years (EU Directive 2006/100/EC) [19]. Dutch EPs need the longer training program and simultaneously the recognition as a Medical Specialist to run the ED as a closed format. Undoubtedly, EPs have improved the quality of patient care on an individual patient level, but until the above-mentioned criteria are met, it will be difficult for EPs to implement overall clinical audits for all ED patients.

This study might also suggest that increasing the size of EDs in The Netherlands could potentially improve quality registrations and meetings. Although audit and feedback were found to have a moderately positive effect in the most recent Cochrane review, this study cannot identify causal effects [20]. Consequently, this would then lead to a reduction in the numbers of EDs. We already see a similar occurrence in GP practices where larger practices seem to have more safety features present [21,22].

### Limitations

Some limitations of this study should be mentioned. We cannot rule out a possible selection bias, as the response rate was 67%. However, our sample included a variety of EDs from every region in the country, suggesting reasonable representativeness.

Respondents had different backgrounds and positions, which may have influenced their answers and could have resulted in information and recall bias. This response rate is however high compared to many surveys among healthcare providers. Furthermore, the relation between non-response and selection bias is not so obvious.

This study documented the presence of EPs in the ED, not taking into account the total numbers of EPs and whether or not they were present 24/7. Therefore we cannot say that it is the influence of the EPs alone that leads to a higher percentage of courses attended [5]. It could well be that it is in fact the hospital that wants to improve the quality of care and therefore employs EPs and lets other physicians, working in the ED, attend necessary courses. However, finding a significant difference in having a radiology meeting in EDs with EPs, an initiative strongly supported by emergency physicians may suggest a positive influence of EPs.

Our study focused on general features, continuing professional education and clinical audit activities, which do not necessarily reflect the quality of patient care. However, after the timeframe of the data collection of this study, the Dutch inspectorate of health issued a document stating that all physicians working in the ED are obligated to attend an ABCDE course before treating ED patients. This might suggest that training and quality activities may benefit the quality of patient care. Given the cross-sectional design, however, this study cannot identify causal effects.

## Conclusion

Our study was designed to examine whether, in the short time that EPs have been present in Dutch EDs, there has been a difference in EDs with and without EPs. It showed that EPs are significantly more present in larger EDs and in EDs where there is more continuing professional education and where there are more clinical audit activities. We assume that these courses and registrations have a positive influence on the quality of care provided in Dutch EDs. Although this study could not identify causal effects, our findings might suggest that the presence of emergency physicians is positively associated with quality of care.

## Competing interests

The authors declare that they have no competing interests.

## Authors’ contributions

All authors participated in setting up the study design, analyzing the data and writing the article. WT gathered the data. WT, PG and MW designed the questionnaire. JK performed the regression analysis. All authors read and approved the final manuscript.
